# A Simple and Rapid Gene Disruption Strategy in *Mycobacterium abscessus*: On the Design and Application of Glycopeptidolipid Mutants

**DOI:** 10.3389/fcimb.2018.00069

**Published:** 2018-03-14

**Authors:** Albertus Viljoen, Ana Victoria Gutiérrez, Christian Dupont, Eric Ghigo, Laurent Kremer

**Affiliations:** ^1^Centre National de la Recherche Scientifique UMR 9004, Institut de Recherche en Infectiologie de Montpellier, Université de Montpellier, Montpellier, France; ^2^Unité de Recherche Microbes, Evolution, Phylogeny and Infection (MEPHI), Institut Hospitalier Universitaire Méditerranée-Infection, Marseille, France; ^3^Centre National de la Recherche Scientifique, Marseille, France; ^4^IRIM, 34293, Institut National de la Santé et de la Recherche Médicale, Montpellier, France

**Keywords:** gene disruption, *Mycobacterium abscessus*, zebrafish, virulence, glycopeptidolipid

## Abstract

Little is known about the disease-causing genetic determinants that are used by *Mycobacterium abscessus*, increasingly acknowledged as an important emerging pathogen, notably in cystic fibrosis. The presence or absence of surface exposed glycopeptidolipids (GPL) conditions the smooth (S) or rough (R) *M. abscessus* subsp. *abscessus* (*M. abscessus*) variants, respectively, which are characterized by distinct infective programs. However, only a handful of successful gene knock-out and conditional mutants have been reported in *M. abscessus*, testifying that genetic manipulation of this mycobacterium is difficult. To facilitate gene disruption and generation of conditional mutants in *M. abscessus*, we have designed a one-step single cross-over system that allows the rapid and simple generation of such mutants. Cloning of as small as 300 bp of the target gene allows for efficient homologous recombination to occur without additional exogenous recombination-promoting factors. The presence of tdTomato on the plasmids allows easily sifting out the large background of mutants spontaneously resistant to antibiotics. Using this strategy in the S genetic background and the target gene *mmpL4a*, necessary for GPL synthesis and transport, nearly 100% of red fluorescent clones exhibited a rough morphotype and lost GPL on the surface, suggesting that most red fluorescent colonies obtained after transformation incorporated the plasmid through homologous recombination into the chromosome. This system was further exploited to generate another strain with reduced GPL levels to explore how the presence of these cell wall-associated glycolipids influences *M. abscessus* hydrophobicity as well as virulence in the zebrafish model of infection. This mutant exhibited a more pronounced killing phenotype in zebrafish embryos compared to its S progenitor and this effect correlated with the production of abscesses in the central nervous system. Overall, these results suggest that the near-complete absence of GPL on the bacterial surface is a necessary condition for optimal pathogenesis of this mycobacterium. They also suggest that GPL content affects hydrophobicity of *M. abscessus*, potentially altering the aerosol transmission, which is of particular importance from an epidemiological and clinical perspective.

## Introduction

*Mycobacterium abscessus* subsp. *abscessus* (*M. abscessus*) is an emerging pathogen increasingly recognized as a serious threat to cystic fibrosis (CF) patients who show a marked vulnerability to infections with this bacterium, which is exacerbated by *M. abscessus*'s extraordinary intrinsic tolerance toward many antibiotics (van Dorn, 2017). Notably, *M. abscessus* is phenotypically resistant to most antitubercular drugs (Nessar et al., 2012). Despite being a member of the rapid-growing environmental phylogeny of mycobacteria, which contain at worst some opportunistic pathogens, *M. abscessus* is considered as a true pathogen that can cause difficult-to-manage, persistent and even deadly infections in otherwise healthy individuals (Varghese et al., 2012; Jeong et al., 2017). It is clear that novel strategies are urgently needed in the fight against *M. abscessus*-induced diseases (van Dorn, 2017).

New approaches to combat *M. abscessus* infections may rely on the discovery of completely new chemical entities that inhibit essential pathways in this bacterium (Viljoen et al., 2017). This may involve the improvement of available compounds to better inhibit putative molecular targets in *M. abscessus*, which may have slightly different structures or functions compared to orthologs efficiently inhibited in other bacteria. It may also involve the discovery of molecules that act in synergy or render efficient available drugs used to treat mycobacterial infections (Singh et al., 2014; Kaushik et al., 2015; Lefebvre et al., 2017). Whatever the case, the development of new anti-*M. abscessus* drugs would benefit immensely from information on the genetic and biochemical vulnerabilities of this bacterium, information which at the current time is very cryptic. Although *M. abscessus* shares many virulence features of *M. tuberculosis* (Ripoll et al., 2009; Choo et al., 2014), recent studies have highlighted common genetic vulnerabilities and drug resistance mechanisms between the two bacteria involving unrelated compounds (Dupont et al., 2016; Halloum et al., 2017; Kozikowski et al., 2017). These data further warrant studies aimed at deciphering the physiology of *M. abscessus* from the genetic level upwards. Information provided by such studies would likely bring answers to questions such as why *M. abscessus* is so resistant to such a large variety of drugs and why, despite being an environmental mycobacterium, it is capable of causing serious infections in humans. Indeed, recent years have seen the development of genetic tools to inactivate *M. abscessus* genes in a site-specific manner (Medjahed and Reyrat, 2009; Halloum et al., 2016; Gregoire et al., 2017; Rominski et al., 2017) as well as to generate conditional gene expression mutants (Cortes et al., 2011). This has led to the identification of a yet small number of genes that are downright essential for virulence or intracellular survival (Bernut et al., 2014, 2016; Halloum et al., 2016) or playing more important roles during certain stages of infection, such as the establishment of infection (Bakala N'Goma et al., 2015) or during the chronic stage of infection (Viljoen et al., 2016).

The present study was undertaken to develop a simple and rapid gene-knock-out strategy as an alternative to approaches relying on double homologous recombination particularly suitable for use in *M. abscessus*. We have focused our study on the *mmpS4*-*mmpL4a*-*mmpL4b* gene locus, the products of which participate in the biosynthesis and transport of glycopeptidolipids (GPL) from the bacterial cytosol where they are produced to the outer layers of the cell wall where they are interspersed in the mycomembrane (Deshayes et al., 2010). Indeed, GPL is the major determinant of the smooth colonial morphotype, characteristic of *M. abscessus* environmental forms (Medjahed et al., 2010). Upon the loss of GPL production, *M. abscessus* converts to a rough colonial morphology and loses attributes that are reminiscent of environmental mycobacteria, such as the ability to form biofilms and sliding motility (Howard et al., 2006).

The availability of new suitable genetic tools described here should undoubtedly further facilitate and stimulate the characterization of new virulence factors and/or the discovery of new drug targets in this emerging pathogen.

## Materials and methods

### Reagents and growth conditions

Unless otherwise stated, all reagents were from Sigma Aldrich. *Escherichia coli* XL1-blue was used for cloning and was routinely maintained at 37°C with shaking at 100–250 rpm. *M. abscessus* CIP104536 was grown in Sauton's broth medium (0.5 g/L K_2_HPO_4_, 0.5 g/L MgSO_4_, 4 g/L L-asparagine monohydrate, 0.05 g/L ferric ammonium citrate, 2 g/L citric acid, 6 % (v/v) glycerol, 1 mg/L ZnSO_4_), Middlebrook 7H9 (Becton Dickinson) or LB broth; and on agar using LB agar. Both liquid and solid media were, in some instances, supplemented with oleic acid/albumin/dextrose/oleic acid (OADC) enrichment or 0.2% (w/v) glycerol and with the detergent tyloxapol at 0.025% (v/v). Antibiotics used to propagate plasmids in *E. coli* were hygromycin (75 μg/mL), kanamycin (25 μg/mL) and zeocine (25 μg/mL). The concentration of kanamycin used to select *M. abscessus* transformants was 250 μg/mL, but was dropped to 100 μg/mL to maintain the plasmids once integration by homologous recombination was confirmed.

For complementation studies, *mmpL4a*::pUX1 transformed with the pMV306-zeo plasmids was cultured in liquid broth and on LB plates containing 50 μg/mL kanamycin and 25 μg/mL zeocine. In order to investigate the rate at which reversion to a wild-type genotype occurred in *mmpL4a*::pUX1 mutants, logarithmic phase cultures of the three mutants (generated with pUX1 plasmids containing varying sizes of the *mmpL4* insert) were first collected by centrifugation and washed three times with PBS. These bacteria were then used to inoculate fresh cultures with and without the antibiotic kanamycin to an OD_600_ = 0.01. The cultures were incubated at 37°C for with slow shaking for 7 days until early stationary phase was reached (OD_600_ > 1). Subsequently, they were used to inoculate fresh medium with or without kanamycin to an OD_600_ = 0.01 and the culturing, sub-culturing process was repeated five more times. After each passage, with and without kanamycin, an aliquot was taken and diluted appropriately in order to view approximately 10,000 colonies by spreading out 100 μL of the diluted suspension on LB agar plates (non-supplemented with kanamycin).

### Construct generation

All specific oligonucleotides and plasmids produced in this study are listed in Table 1. All cloned fragments were amplified using purified *M. abscessus* genomic DNA and Phusion polymerase (Finnzymes). To produce the backbone vector used to generate single cross-over gene disruptions, the NheI restriction site in the vector pMV261 (Stover et al., 1991) was first replaced with an AflII site by self-ligating the T4 polynucleotide kinase (NEB) phosphorylated PCR product obtained with the primer set pMV3′ and pMV(NheI-AflII) and pMV261 as template. Subsequently, the obtained plasmid (pMV261-AflII) was digested with XmnI and ligated to the T4 DNA polymerase (Invitrogen) blunted SpeI fragment of pTEC27 containing the tdTomato and hygromycin resistance cassettes (Takaki et al., 2013). The resulting construct, pUX1, contained neither a mycobacterial origin of replication nor features necessary for integration into the mycobacterial chromosome. For simple cloning into pUX1, it contains the convenient restriction enzymes BamHI, AflII, PvuII, NheI, and SpeI, the latter two creating compatible ends, which will also accept XbaI overhangs. pUX2, containing the mWasabi green fluorescent protein gene instead of tdTomato, was obtained in a similar fashion using pTEC15 (Takaki et al., 2013). The plasmid for single cross-over recombination containing the acetamidase promoter, pUX3, was made by replacing the NheI-BamHI fragment of pUX1 containing the hygromycin resistance cassette with the NheI-BamHI fragment of pSD26 (Daugelat et al., 2003) containing the acetamidase promoter.

**Table 1:** Oligonucleotides and plasmids generated and used in the study.

**Name**	**Sequence/Description**
***OLIGONUCLEOTIDES***	
pMV3′	GCCTGGCAGTCGATCGTACG
pUX1-NheI	ACGGCATGGACGAGCTGTAC
pMV(NheI-AflII)^a^	CCGCGGTGATCAGCTTAAGCCAACAAAGCGAC
mmpL4a_Fw_SpeI	TGTGACTAGTCAGATGGGGAAGGTCTTTCA
mmpL4a_Rev_BamHI_100	CAAGAAGGATCCCGTAATACTTGTGCGCATCGTCTCC
mmpL4a_Rev_BamHI_500	GAGAGGATCCCGGTGAACAACAGGATGATG
mmpL4a_Rev_BamHI_1000	GAGAGGATCCCGGTGTAGCTGGGGTTGTAT
mmpL4a_Rev_BamHI_1500	GTGTGGATCCTGTTTGAGCATGTCGTCCAT
mmpL4a_conf_left	CTTCCGTGGTCCGTCAAAT
mmpL4a_conf_right	CATTCGTGAGACCAGCAACA
mmpS4_Fw_BamHI	GAGTAGGATCCATGCGTCTGTGGATTCCGCTG
mmpS4_Rev_AflII	GAGTACTTAAGCATGATGCTTCCCACCGCG
mmpS4_conf_left	GGATACCCAGTGGCTTGAAA
mmpL4a-mmpL4b_left	TGCGTCTGTGGATTCCGCTG
mmpL4a-mmpL4b_right_HindIII	GAGAGAAAGCTTAGTACGTCATCCCGGTGTTC
***PLASMIDS***	
pMV261	Multicopy *E. coli*- mycobacterium shuttle vector, kan^r^ (Stover et al., 1991)
pMV261-AflII	Variant of pMV261 in which the NheI site of the polylinker was changed to an AflII by site-directed mutagenesis, kan^r^
pMV306-zeo-*mmpL4a*	Integrative *E. coli*- mycobacterium shuttle vector, zeo^r^, contains *M. abscessus* subsp. *abscessus mmpL4a* under the control of the *hsp60* promoter (Bernut et al., 2016)
pUX1	Plasmid produced by ligating the *colE1* origin and kan^r^ cassettes-containing XmnI fragment of pMV261-AflII to the blunted SpeI fragment from pTEC27 (Takaki et al., 2013) containing the tdTomato and hyg^r^ cassettes.
pUX2	Plasmid produced by ligating the *colE1* origin and kan^r^ cassettes-containing XmnI fragment of pMV261-AflII to the blunted SpeI fragment from pTEC15 (Takaki et al., 2013) containing the mWasabi and hyg^r^ cassettes.
pUX3	Plasmid produced by replacing the hyg^r^ cassette flanked by NheI and BamHI sites of pUX1 with the acetamidase inducer elements of pSD26 (Daugelat et al., 2003).

a *Underlined are restriction enzyme recognition sequences*.

In order to produce the pUX1 derivatives to inactivate *mmpL4a*, different size PCR products of a sequence internal to the *mmpL4a* gene was first obtained using the primers mmpL4a_Fw_SpeI and mmpL4a_Rev_BamHI_100, or mmpL4a_Rev_BamHI_500, or mmpL4a_Rev_BamHI_1000, or mmpL4a_Rev_BamHI_1500 (Table 1). These amplicons were subsequently treated with SpeI and BamHI and ligated to NheI-BamHI-linearized pUX1. To generate the pUX2-*mmpL4a*_1500bp derivative, the SpeI-NheI restriction product of pUX2, containing the *oriE* and mWasabi was ligated to the SpeI-XbaI fragment of pUX1-*mmpL4a*_1500bp containing the kanamycin cassette. pMV306-zeo-*mmpL4a*-*mmpL4b* was made by first amplifying the *mmpL4a* and *mmpL4b* operon of *M. abscessus*, using the primers mmpL4a-mmpL4b_left and mmpL4a-mmpL4b_right_HindIII (Table 1). The PCR product was subsequently EcoRI-HindIII digested and ligated to EcoRI-HindIII-linearized pMV306-zeo-*mmpL4a* (Bernut et al., 2016). To generate the pUX3 derivative used to replace the *mmpS4* promoter with the acetamidase promoter and regulatory elements of *M. smegmatis*, a 300 bp 5′-portion of the *mmpS4* gene was first amplified by PCR using the primers mmpS4_Fw_BamHI mmpS4_Rev_AflII (Table 1), subsequently digested with BamHI and AflII and ligated to BamHI-AflII-linearised pUX3.

### Preparation of electrocompetent mycobacteria and transformation

To obtain highly electrocompetent *M. abscessus*, a single colony was used to inoculate Sauton's medium supplemented with OADC and tyloxapol (Sauton^OADC^). This culture was grown at 37°C with gentle agitation (<60 rpm) until an OD_600_ of about 5 was reached (in the case of the R variant, bacteria were grown for a similar duration and then concentrated to about 5 OD_600_), then aliquoted into 1 mL volumes and frozen at −80°C. A single such aliquot was thawed and used to inoculate 200 mL Sauton^OADC^ in an Erlenmeyer flask, which was shaken overnight at 100 rpm and 37°C until an OD_600_ reading of approximately 0.8 was achieved. Bacteria were then chilled on icy water for 1–2 h, collected by centrifugation (3,000 × g, 15 min, 4°C) and washed four times with gradually decreasing volumes (50, 25, 10, and 5 mL) of ice-cold wash solution (10% glycerol (v/v), 0.025% tyloxapol). Electrocompetent bacteria were resuspended in 1 mL wash solution and, in the case of the R variant, deaggregated by 15 passages through a 26 GA syringe needle. For high yield of transformants, 1–10 μg plasmid DNA were added to 200 μl of fresh electrocompetent bacilli, which were then transferred to a chilled 0.2 cm electrode gap GenePulser eletroporation cuvette (Bio-Rad) and subjected to electrotransformation using a GenePulser Cxell electroporator (Bio-Rad) and the following settings: 2.5 kV, 1,000 Ω and 25 μF. Electroporated bacteria were recovered in 1 mL ice cold Sauton^OADC^, transferred to 15 mL centrifuge tubes and incubated for 2–16 hrs at 37°C with gentle shaking. The bacteria were then plated out on LB agar plates containing the appropriate antibiotic and incubated at 37°C until colonies appeared (around 3–5 days later). Using this protocol, we routinely obtained efficiencies of 2–5 × 10^4^ CFU/μg DNA transformed and 10–100 red fluorescent single cross-over colonies. Cryogenic storage of electrocompetent bacteria at −80°C, preceded by snap freezing of cells on liquid nitrogen, resulted in about a log decrease in electro-transformation efficiency, which was still high enough to obtain single cross-over red fluorescent rough colonies, but at a lower frequency.

### GPL extraction and analysis

GPL extraction was performed as reported previously (Villeneuve et al., 2003) with some modifications. In brief, bacteria were cultured to Log growth phase (OD_600_, 0.6–1.2) in 7H9^OADC^ (30 mL) without agitation and without detergent. After centrifugation, lipids were extracted from the bacterial pellets first with chloroform/methanol (1:2, v/v) and then three times by chloroform/methanol (1:1, v/v). Lipid extracts were pooled and dried under a stream of nitrogen prior to resuspension in chloroform. Lipids were then washed at least four times with a volume of H_2_O equal to the volume of chloroform, dried, re-dissolved in dichloromethane and subjected to thin layer chromatography (TLC) analysis using Silica gel 60 F_254_ plates (Merck). GPLs were separated using chloroform/methanol (9:1, v/v), sprayed with orcinol/sulphuric acid vapor prior to revelation by charring.

### Hexadecane partitioning

Briefly exponentially growing mycobacteria were collected by centrifugation and washed twice with PUM (100 mM K_2_HPO_4_, 54 mM KH_2_PO_4_, 30 mM urea, 0.8 mM MgCl_2_) buffer prior to carrying out hexadecane partitioning, as previously described (Jankute et al., 2017).

### Zebrafish care and ethics statements

All zebrafish experiments were approved by the Direction Sanitaire et Vétérinaire de l'Hérault et Comité d'Ethique pour l'Expérimentation Animale de la région Languedoc Roussillon under the reference CEEA-LR-1145. Experiments were done using the *golden* mutant (Lamason et al., 2005) crossed with wild-type AB zebrafish, maintained as described earlier (Bernut et al., 2014). Ages of embryos are expressed as hours post fertilization (hpf).

### Zebrafish embryo infections

The various *M. abscessus* strains expressing tdTomato were prepared, injected and monitored according to protocols reported previously (Bernut et al., 2015). In brief, infections were performed by microinjection of bacterial suspensions (≈2 nL containing 150–200 bacteria) intravenously in dechorionated and anesthetized 30 hpf embryos. The size of the inoculum was verified *a posteriori* by injecting 2 nL of each bacterial suspension in sterile PBS^T^ and plating on 7H10^OADC^. For kill kinetics, infected larvae were transferred into 12 well plates (3 embryos/well) and incubated at 28.5°C. Survival curves were drawn by counting dead embryos every day for up to 13 days. To analyze abscess size induced by different strains of *M. abscessus*, the largest abscess in each infected fish was first marked off on scale-set microscope images of whole embryos using the polygon selection tool of the Fiji software. Next, the measure function was used in order to obtain abscess area in μm^2^.

## Results

### Efficacy of single cross-over homologous recombination at the GPL locus

**Figure 1:**
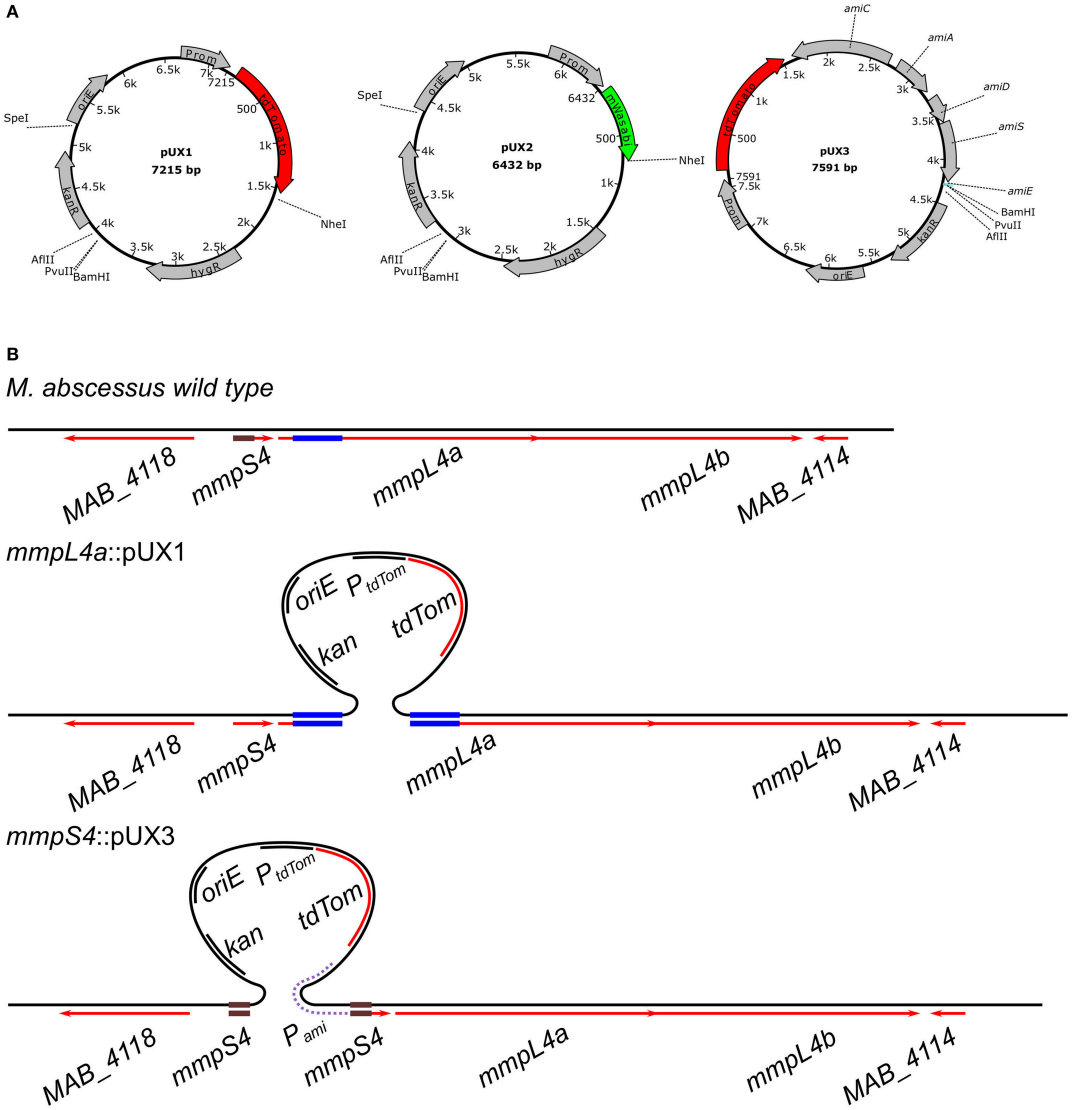
The principal of single cross-over gene disruption. **(A)** Maps of pUX1, pUX2 and pUX3. **(B)** The GPL-transporter locus, consisting of *mmpS4, mmpL4a* and *mmpL4b* in *M. abscessus* (Top). Disruption of *mmpL4a* through integration of pUX1-*mmpL4a* (Middle). Replacement of the *mmpS4*-*mmpL4a*-*mmpL4b* operon promoter with an acetamide-inducible promoter through integration of pUX3 (bottom).

In this simple single cross-over strategy, a PCR amplicon of a region within the target gene is cloned into pUX1 or pUX2 (Figure 1A), neither of which contain a mycobacterial origin of replication nor the integrase and *attP* site necessary for integration into the mycobacterial chromosome. Once *M. abscessus* is electro-transformed with such a plasmid, the sole means by which this plasmid could be propagated within the dividing bacteria is by inserting itself into the bacterial chromosome through homologous recombination with the target gene, resulting in specific gene disruption, as depicted in Figure 1B. We further reasoned that owing to the high rate of spontaneous resistance to selective antibiotics seen for *M. abscessus*, the presence of a brightly fluorescing marker, such as tdTomato (red) or mWasabi (green), on the plasmid may largely facilitate subsequent screening of electro-transformed putative knock-out colonies. To make the cloning step of the internal gene fragment easy, the backbones of pUX1 and pUX2 (Figure 1B) were designed to contain the convenient restriction sites for BamHI, AflII and PvuII flanked by kanamycin and hygromycin cassettes. On the opposite side of the kanamycin and hygromycin cassettes lie the NheI and SpeI restriction sites, allowing replacement of one of the resistance cassettes with the cloned target gene sequence.

**Figure 2:**
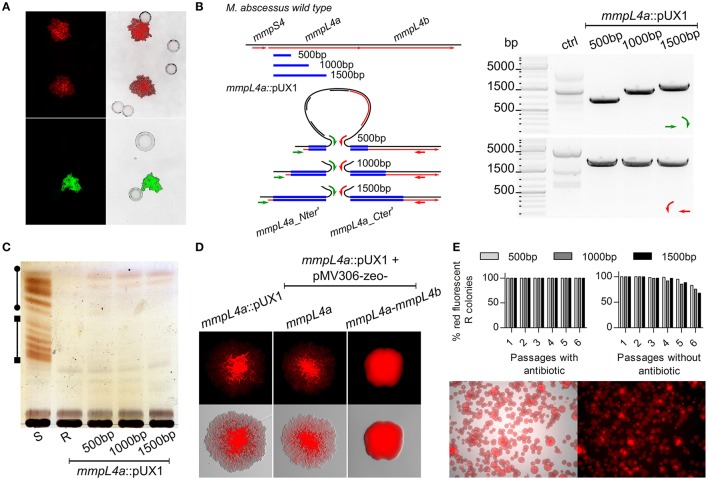
Single cross-over gene disruption of *mmpL4a*. **(A)** Representative fluorescence and overlay images showing the selection of red fluorescent colonies with exclusively a R morphotype after transformation of *M. abscessus* S with pUX1-*mmpL4a* (Top) or pUX2-*mmpL4a* (Bottom). Colonies arising as a result of spontaneous resistance to the selective antibiotic are non-fluorescent and smooth. **(B)** PCR screening and sequencing strategy employed to easily confirm correct integration of the pUX1 plasmid into the target gene, *mmpL4a*. Expected amplicon size generated with the green primer set are 922 bp (*mmpL4a*::pUX1_500bp), 1462 bp (*mmpL4a*::pUX1_1000bp) and 1790 bp (*mmpL4a*::pUX1_1500bp), while the expected size for the red primer amplicon is 2444 bp (the same size for all three mutants). **(C)** TLC showing disappearance of glycopeptidolipids (GPL) in *mmpL4a*::pUX1 mutants. The line with circle caps indicate diglycosylated GPL, while the line with the square caps indicate triglycosylated GPL. **(D)** Representative fluorescence and overlay images showing that complementation of *mmpL4a*::pUX1 with *mmpL4a* alone does not restore a S colony morphology while complementation with *mmpL4a* and *mmpL4b* does. **(E)** Stability of pUX1 integration into the target gene *mmpL4a* was assessed by passaging *mmpL4a*::pUX1 (500, 1,000, and 1,500 bp insert) strains in the presence (top left) or absence (top right) of selective kanamycin, followed by serially diluting aliquots and plating these on LB agar without kanamycin in order to observe up to 10,000 single colonies. Bottom. Representative fluorescence and composite images showing purely rough red fluorescent colonies of *mmpL4a* after two passages in liquid medium in the absence of selective kanamycin.

We chose to exploit the *mmpL4a* gene that encodes one of the constituents of the GPL biosynthesis/transport apparatus, as recently demonstrated in *Mycobacterium abscessus* subsp. *bolletii* (Bernut et al., 2016), as a target gene. Knock-outs of this gene would be easily tractable after transformation due to their distinct R colony morphology clearly setting them apart from S colonies that appear either due to spontaneous resistance or illegitimate recombinational uptake of transformed plasmid. Since smaller sizes of cloned fragment may be less prone to homologous recombination, which could be particularly problematic when smaller genes are targeted, we started by cloning internal fragments of *mmpL4a* ranging from 100–1,500 bp in length. As detailed in Table 2, with an electroporation efficiency just exceeding 10^4^ CFU/μg plasmid, which is more than a log lower usually obtained with *M. smegmatis* mc^2^155 (Parish and Stoker, 1998), transforming the same preparation of electrocompetent *M. abscessus* S with pUX1-*mmpL4a* yielded an unexpectedly large number of red-fluorescent colonies. Importantly, all these red-fluorescent colonies appeared with a distinct R morphology, while spontaneous resistant colonies presented the clear S morphology of the progenitor S strain (Figure 2A). Screening of several randomly chosen R and red fluorescent colonies using appropriate PCR/sequencing confirmed the presence of pUX1 within the chromosomal *mmpL4a* locus in all selected clones (Figure 2B). This indicates that uptake of pUX1-*mmpL4a* occurred exclusively through homologous recombination of the plasmid with the chromosomal *mmpL4a* locus. Indeed, no red-fluorescent kanamycin resistant *M. abscessus* colonies could be obtained by transformation with the empty pUX1 despite several attempts (data not shown). These results suggest (i) that homologous recombination occurs at a higher frequency in *M. abscessus* than that reported for other commonly investigated mycobacterial species, such as *M. smegmatis* (Pavelka and Jacobs, 1999), *M. bovis* BCG (Sander et al., 2001) and *M. tuberculosis* (Parish et al., 1999) and (ii) that illegitimate recombination events between pUX1 and the *M. abscessus* chromosome occurs at a very low level or not at all. As expected, the frequency of red fluorescent R colonies obtained with pUX1-*mmpL4a* containing a 1,500 bp cloned fragment of *mmpL4a* was much greater than the frequency obtained with constructs carrying smaller sizes (500 bp) of the cloned fragment (Table 2). After several attempts, we failed to isolate a single R red fluorescent colony from bacteria transformed with pUX1-*mmpL4a* containing a 100 bp cloned-fragment of the gene (Table 2). To test the vector pUX2, which is essentially exactly the same as pUX1 except that it contains mWasabi instead of tdTomato, we made a version of this vector containing the 1500 bp *mmpL4a* insert as for pUX1-*mmpL4a*_1500bp. As anticipated, transforming *M. abscessus* with this plasmid yielded green fluorescent R colonies (Figure 2A).

**Table 2:** Frequency of plasmid uptake by homologous recombination.

**Size of cloned fragment in pUX1/3 (bp)**	**100^b^**	**300^c^**	**500^b^**	**1,000^b^**	**1,500^b^**
N° Red fluorescent colonies	0	3	5	43	147
% Colonies PCR positive	0	100	100	100	100
% Efficiency^a^	0	100	100	100	100
Transformation efficiency	1.45 × 10^4^ CFU/μg DNA				

a *Percentage of total number of colonies that are red fluorescent and PCR positive*.

b *Cloned into pUX1*.

c *Cloned into pUX3*.

### Mutants generated by single cross-over homologous recombination exhibit expected phenotypes

S and R morphotypes are often associated with the presence or absence, respectively, of GPL in mycobacteria (Billman-Jacobe et al., 1999; Eckstein et al., 2000; Recht and Kolter, 2001), including *M. abscessus* and *M. abscessus* subsp. *bolletii* (Howard et al., 2006; Bernut et al., 2016). This prompted us to analyze the GPL profile in the *mmpL4a::*pUX1 mutants by thin-layer chromatography (TLC). As anticipated, and similarly to the *M. abscessus* CIP104526 R type strain, all the mutated R fluorescent mutants were characterized by the loss of GPL production (Figure 2C). Abrogation of GPL production/transport occurred in all mutants generated with the pUX1 constructs harboring different sizes of the *mmpL4a* fragment. We next aimed to complement the *mmpL4a*::pUX1 mutant by transforming *mmpL4a*::pUX1_500bp with the pMV306-zeo-*mmpL4a* plasmid (Figure 2D). Re-introduction of a copy of *mmpL4a* on the integrative vector did not confer a S colony morphology. It was thus possible that integration of pUX1 into the *mmpL4a* gene had a polar effect also on *mmpL4b*, which is located downstream of *mmpL4a* in a possible operon configuration. We thus constructed a new plasmid, pMV306-zeo-*mmpL4a*-*mmpL4b*, and introduced it in the mutant. Re-introduction of both *mmpL4a* and *mmpL4b* fully restored the wild-type S colony morphology in the *mmpL4a*::pUX1_500bp mutant (Figure 2D), proving that pUX1 exerted a polar effect on the expression of a gene downstream in the operon of the targeted gene for mutation.

Since homologous integration of pUX1-*mmpL4a* into the chromosome resulted in the duplication of the central cloned gene fragment (Figure 1B), it is conceivable that by a second homologous recombination event between the 5′ and 3′ truncated parts of the *mmpL4a* gene with the pUX1 insertion, pUX1-*mmpL4a* could be lost from the chromosome, hence resulting in reversion to the wild-type genotype. We thus assayed the possibility of such a reversion to occur by serially sub-culturing the different *mmpL4a*::pUX1 mutants in the presence or absence of the selective antibiotic kanamycin, preparing serial dilutions of the cultures after each passage and plating these out on LB agar so as to observe single colonies after 4–5 days of incubation. Reversion to the wild-type phenotype could be easily observed in the loss of R morphology and red fluorescence. After six serial passages in the presence of kanamycin not a single S, non-fluorescent colony could be observed among at least 10,000 colonies. In the absence of kanamycin, no wild-type reversions were observed after two passages. However, after a third passage, between 1 and 3% of colonies observed were S and non-fluorescent and, after six passages, this percentage had increased to between 15 and 35% of the total number of colonies observed (Figure 2E). In addition, the rate of reversion appeared to be directly related to the size in nucleotide bases of the cloned fragment in pUX1. These data attest to the stability of pUX1 integration in the presence of selective antibiotic and the relative stability even in the absence of antibiotic pressure.

Taken together, these results show that pUX1-based single cross-over targeted inactivation of genes is simple and occurs at a high frequency in *M. abscessus*.

### Generation of a low-level GPL producing rough variant

Next, we aimed to extend the single cross-over strategy to generation of conditional mutants. A derivative of pUX1, designated pUX3, that contains the promoter and a truncated portion of the coding sequence of the *M. smegmatis amiE* gene (Parish et al., 1997) as well as the acetamide responsive elements, *amiA, amiC, amiD*, and *amiS* was constructed (Figure 1A). A polylinker within the truncated *amiE* coding sequence allows simple cloning of a 5′ fragment of a gene of interest in frame with the *amiE* coding sequence. Subsequent transformation of bacteria allowing homologous recombination between the gene of interest and pUX3 results in replacement of the native gene promoter with the acetamide responsive elements of pUX3 (Figure 1B).

**Figure 3:**
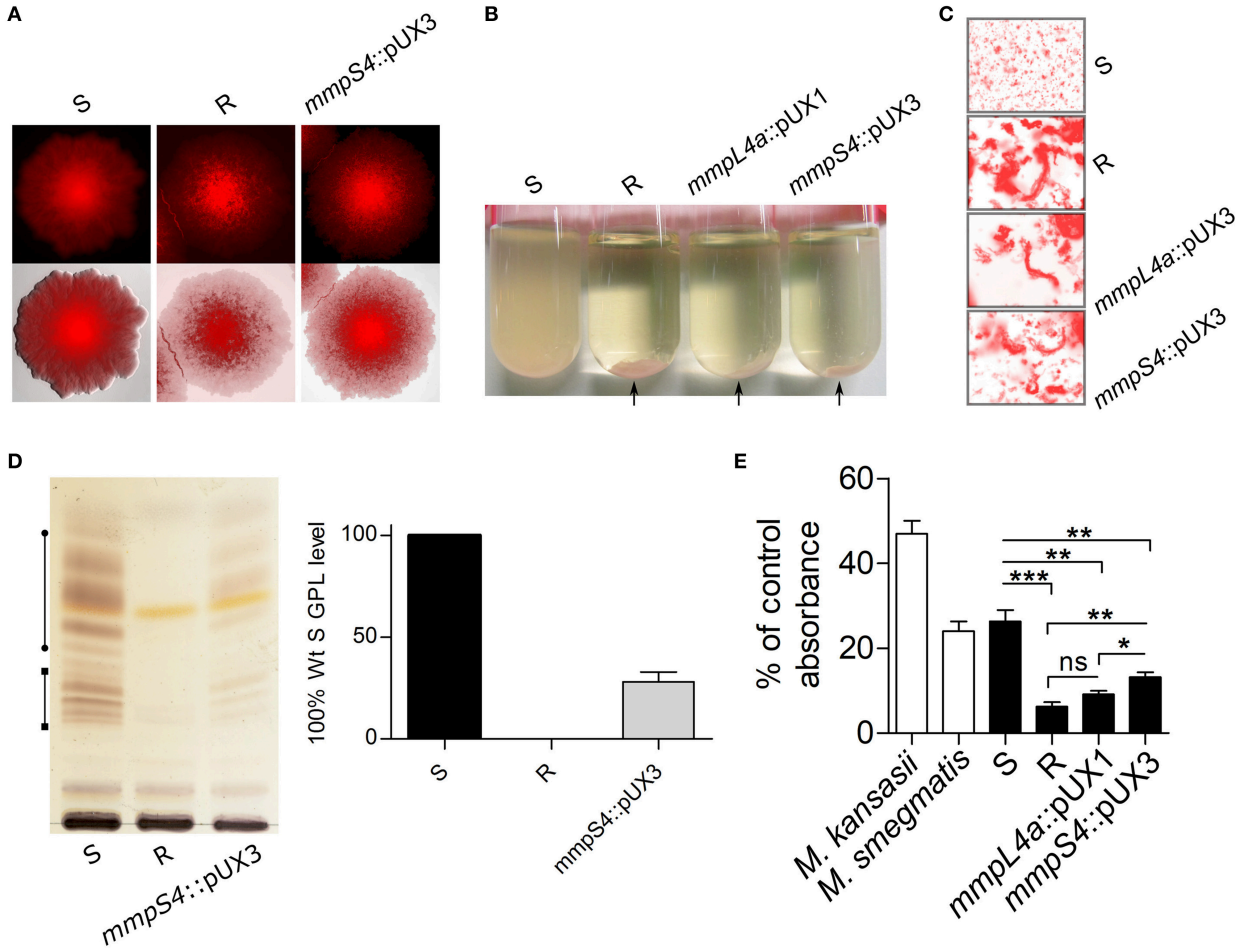
Single cross-over promoter replacement of *mmpS4*. **(A)** Representative bright field, fluorescence and composite images showing the rough colony morphology associated with the *mmpS4*::pUX3 strain. **(B)** Like the R, but unlike the S variant, *mmpS4*::pUX3 sediments rapidly in liquid culture. Exponentially growing cultures (OD_600_ = 1) were transferred to glass tubes and allowed to sediment for 10 min before pictures were taken. Arrows indicate the sedimented bacterial aggregates. **(C)** Representative fluorescence images showing serpentine cords in broth culture of *M. abscessus* R, *mmpL4a*::pUX1 and *mmpS4*::pUX3 but not of *M. abscessus* S. **(D)** Left: Low-level GPL production is maintained in *mmpS4*::pUX3 as assessed by TLC, despite its rough colonial phenotype and aggregative properties. The line with circle caps indicate diglycosylated GPL, while the line with the square caps indicate triglycosylated GPL. Right: TLCs produced as in the left from four independent experiments were subjected to a densitometric analysis in order to determine the relative level of GPL present in the *mmpS4*::pUX3 mutant. Shown is the mean and standard deviation calculated from these experiments. **(E)** *M. abscessus* R is more hydrophobic than S, while the *mmpS4*::pUX3 mutant strain's hydrophobicity is between S and R, as assessed by hexadecane partitioning and shown as hydrophobicity index (in percentage of aqueous phase OD prior to partitioning). Histograms and error bars are means and standard deviations of at least three independent experiments. Differences between means were analyzed for significance using a two-tailed Student's *T*-test employing Welch's correction for unequal variances. Ns, non-significant, ^*^p < 0.05, ^**^p < 0.01, ^***^p < 0.001.

To test the efficacy of the pUX3 acetamide-inducible conditional mutant system, we again focused on the locus in *M. abscessus* that is responsible for GPL export, consisting of the genes *mmpS4, mmpL4a*, and *mmpL4b*. Since these three genes are arranged in an operon configuration and, hence, it is likely that the three genes are transcribed as a single mRNA transcript, a 300 bp fragment of the *mmpS4* gene was cloned into pUX3 (Figure 1B). Transformation of the *M. abscessus* S variant with this plasmid, pUX3-*mmpS4*, yielded red-fluorescent colonies with a rough morphology (Figure 3A). PCR/sequencing analysis confirmed the proper replacement of the *mmpS4* promoter with the acetamidase elements of pUX3 (data not shown). Moreover, the *mmpS4*::pUX3 mutant, like the R reference strain, aggregated in liquid culture and failed to produce homogenous suspensions that typify *M. abscessus* S cultures (Figure 3B). Importantly, serpentine cords, a hallmark of *M. abscessus* R virulence (Bernut et al., 2014), were observed in liquid cultures of *mmpS4*::pUX3 as for *mmpL4a*::pUX1 and *M. abscessus* R, but not for *M. abscessus* S (Figure 3C).

Several tests employing acetamide as supplement were then performed in order to induce MmpS4-MmpL4a-MmpL4b expression and GPL production both in broth and on solid agar culture. However, stable high-level GPL production supporting S phenotypes was not fully achieved, despite testing various acetamide concentrations and medium compositions (data not shown). In addition to colony morphology and bacterial aggregation phenotypes, GPL profiles of cultures were also assessed by TLC. As shown in Figure 3D, the *mmpS4*::pUX3 strain still produced low levels of GPL compared to the S progenitor (approximately 30% of S levels), even in the absence of inducer. Therefore, despite our inability to generate a strain for which GPL production and the R-to-S transition could be conditionally induced by the presence or absence of an inducer (acetamide), we ascertained a new *M. abscessus* mutant that behaved like the R variant, aggregating in culture and producing rough and dry colonies, but still producing and exporting low levels of GPL.

### GPL loss is a determinant of mycobacterial hydrophobicity

Partitioning of mycobacterial cultures between hexadecane and an aqueous buffer has recently been used as a quantitative marker of hydrophobicity in *Mycobacterium tuberculosis* evolution and pathogenicity, ranging from hydrophilic environmental low-pathogenicity ancestors *Mycobacterium kansasii* and *Mycobacterium canettii* to highly hydrophobic virulent tubercle bacilli (Minnikin et al., 2015; Jankute et al., 2017). Given the important difference in the morphotype and aggregative properties characterizing the *M. abscessus* variants, hexadecane-aqueous buffer partitioning was applied to the S, R and the different *M. abscessus* mutants generated in this study to unveil a possible link between hydrophobicity and GPL content. In addition, *M. kansasii* Hauduroy (ATCC12478), included as an internal control strain, was found to be highly hydrophilic, as reported previously (Jankute et al., 2017; Figure 3E). The S variant shared comparable partitioning profiles in hexadecane, with *M. smegmatis* mc^2^155 and both were significantly more hydrophilic than the R variant or the *mmpL4a*::pUX1 mutant. This indicates that lack of the hydrophilic GPL components in the parental R strain or the *mmpL4a*::pUX1 mutant is responsible for the increased hydrophobicity of these strains. Interestingly, the *mmpS4*::pUX3 derivative, which produces low levels of GPL but expresses rough and aggregative properties, exhibited intermediate levels of partitioning and hydrophobic properties as compared to the S and R strains (Figure 3E), further confirming a positive correlation between GPL production and hydrophilicity.

### Low-level GPL production impedes virulence of a rough variant

**Figure 4:**
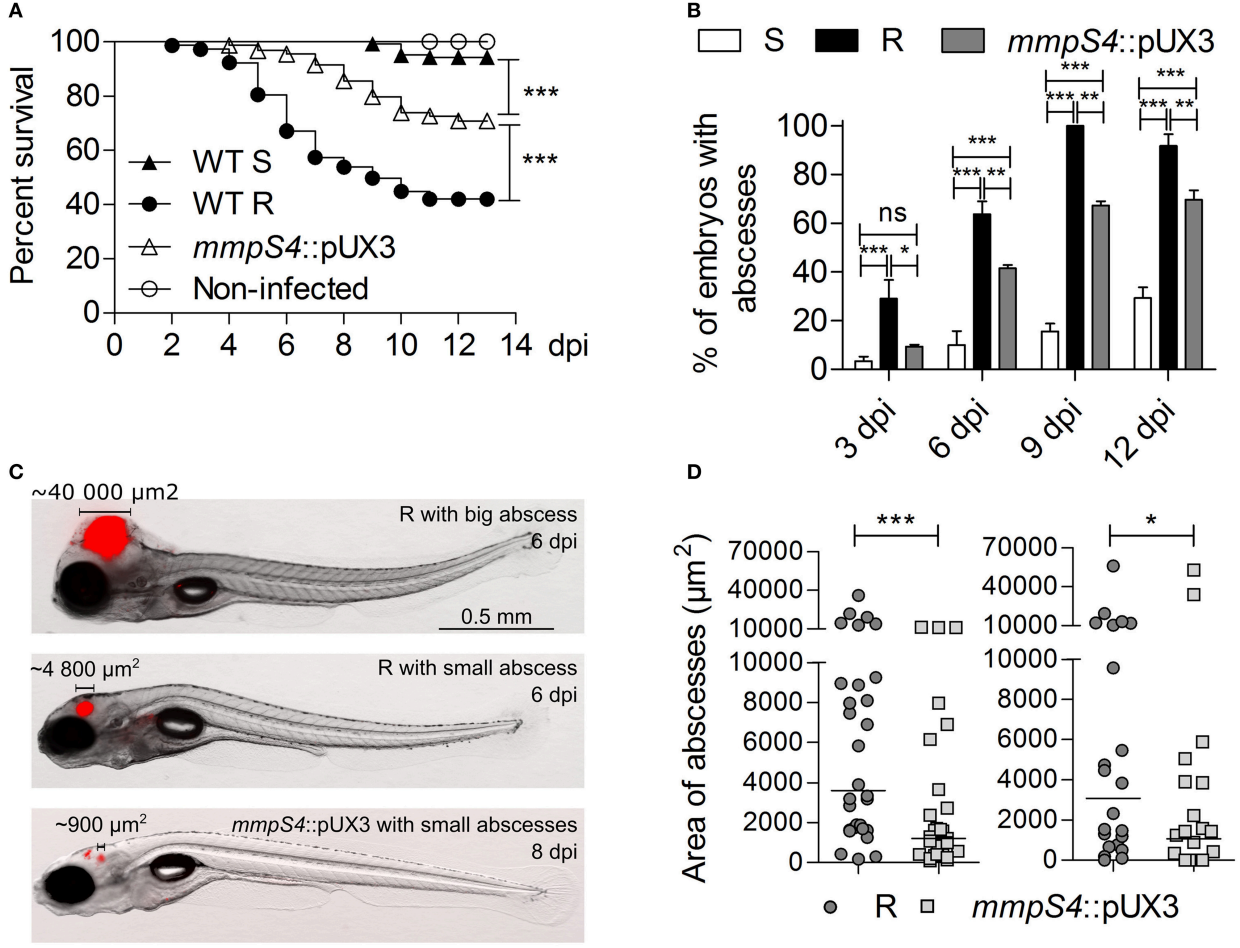
Low-level GPL producing *mmpS4*::pUX3 is more virulent than the S variant in zebrafish embryos, but not as virulent as the R variant. **(A)** Survival curves of zebrafish embryos infected with different strains. Killing of zebrafish embryos over the course of the experiment by different mycobacterial strains were compared using the Mantel-Cox log rank test. Shown is the pooled data from five independent experiments. In each experiment, 25–30 embryos were injected each with 200 ± 50 cfu. **(B)** Kinetic of abscess appearance in the population of infected embryos over a 12-day infection period showing retarded abscess appearance in *mmpS4*::pUX3-infected embryos compared to embryos infected with the R variant. Histograms and error bars represent means and their associated standard errors calculated from two independent experiments using 20 embryos per bacterial strain on each occasion. Data was analyzed by two-way ANOVA and Bonferroni post-tests. Ns, non-significant, ^*^p < 0.05, ^**^p < 0.01, ^***^p < 0.001. **(C)** Representative images of embryos infected with the R variant at 6 dpi and *mmpS4*::pUX3 at 8 dpi showing smaller abscesses in the case of *mmpS4*::pUX3. **(D)** Scatterplots from two independent experiments showing sizes in area of the largest abscess per infected embryo over the 12-days infection period (*n* = 20 per bacterial strain in each experiment). Median abscess area between the R variant and *mmpS4*::pUX3 were statistically compared using a one-tailed Mann Whitney test. ^*^p < 0.05, ^***^p < 0.001.

The zebrafish embryo was recently used as a relevant animal model to describe the chronology of the events leading to acute infection with *M. abscessus* and to compare the increased virulence of the R over the S variant (Bernut et al., 2014, 2015). Herein, we further exploited this model to compare the virulence and the physiopathological symptoms of the parental S and R strains with the *mmpS4*::pUX3 mutant. Around 200 CFU of the tdTomato-expressing strains were injected in the caudal vein of embryos at 30 h post-fertilization (hpf). Mortality was monitored at a daily basis for 13 days. Consistent with previous findings (Bernut et al., 2014, 2016), injection of the S form failed to induce larval mortality whereas intravenous injection of the R variant was associated with a robust infection that led to about 60% of mortality at 10 dpi (Figure 4A). In contrast, the survival curve of embryos infected with *mmpS4*::pUX3 showed an intermediate profile, characterized by a delay in killing as compared to infection with the R strain and with a reduced mortality rate (around 30% at 13 dpi). The transparency of the embryos was next exploited to count and characterize the abscesses, considered as typical pathophysiological markers of severity of the infection in this animal model (Bernut et al., 2014, 2015, 2016). The proportion of embryos infected with the S strain and developing abscesses remains low as compared to the one infected with the R variant, as reported earlier (Bernut et al., 2014, 2015, 2016). However, abscesses were detected in around 60% of the *mmpS4*::pUX3-infected embryos over 9 dpi and this was significantly reduced as compared to the R-infected animals (Figure 4B). In all infected groups, the majority of the abscesses were found within the central nervous system, mainly in the brain (Figure 4C). Careful inspection of the infected embryos unraveled significant differences in the size of the abscesses in the R- or *mmpS4*::pUX3-infected embryos (Figure 4C). A quantitative analysis was done by measuring the area of the abscesses, thus mirroring their size/volume, in individuals of both groups of infected animals. This clearly revealed that the size of the abscesses was highly heterogenous in both the R- and the *mmpS4*::pUX3-infected populations (Figure 4D). However, the median size of the abscesses in the former group was significantly more pronounced than the one in the *mmpS4*::pUX3-infected group. This latter group also contained a large number of embryos with no or very small abscesses (Figures 4C,D).

Overall, these results further support the direct relationship between the GPL content and virulence of *M. abscessus* and that low-level GPL production impedes the induction of the physiopathological symptoms and virulence of a R variant.

## Discussion

In comparison to the large number of studies dedicated to delineating the pathophysiology of *M. tuberculosis*, non-tuberculous mycobacteria (NTM) have been largely neglected as pathological organisms. However, in recent years *M. abscessus*, one such NTM, has been witnessed as a serious and emerging infectious agent, surpassing tuberculosis in many industrialized countries. Not only is it responsible for severe post-surgical and pulmonary infections (Jeong et al., 2017; Koh et al., 2017), but it is also intrinsically resistant to most currently available antibiotics (Medjahed et al., 2010; Nessar et al., 2012). This situation is worsened by the recent whole-genome sequencing studies of *M. abscessus* in CF patients indicating a possible human-to-human transmission of this infection (Bryant et al., 2013, 2016). The treatment duration being very long (lasting for 1–2 years) and associated with multi-drug regimens leading often to disappointing results, it resembles that of XDR-TB. In addition, *M. abscessus* can provoke latent infections and persist silently for many years in lesions before re-emerging to produce an acute infection (Tomashefski et al., 1996; Cullen et al., 2000; Medjahed et al., 2010). Unfortunately, despite its increasing importance as a true pathogen, the lack of efficient genetic methods to easily manipulate *M. abscessus* has contributed to the little knowledge on its genetic requirements to establish acute and chronic infections and to adapt to its various environments and hosts. In this context, the present study was undertaken to provide an alternative to the standard recombineering strategy. It is based on a very simple method to disrupt genes by single homologous recombination occurring between the gene targeted for disruption and a suicide plasmid with a cloned internal fragment of the target gene. Cloning of only a single PCR product is required to generate the single cross-over substrate plasmid, no induction of recombineering proteins is required prior to preparation of electrocompetent cells and the presence of the tdTomato red (or mWasabi green) fluorescent marker on the plasmid eliminates the very high background of spontaneous resistant mutants, thus avoiding fastidious and time-consuming screening procedures. However, careful attention should be paid during design of PCR amplicons for cloning of internal target gene fragments and in the case it is required to clone smaller (< 500 bp) sequences of target genes, several transformation attempts may be required to obtain knock-outs. In addition, use of this technique to disrupt genes found in operons may have undesired polar effects on the genes located downstream of the target gene, although this issue can be subsequently addressed by appropriate complementation studies. Another major advantage of this method is that it avoids the subsequent time-consuming curing procedures of the mutated strains that characterizes the recombineering strategy, which relies on the use of a plasmid carrying the exonucleases from phage Che9c (van Kessel and Hatfull, 2007). In addition, the one-step procedure described here allows to directly integrate a red fluorescent marker into the mutant, which is particularly useful for subsequent *in vivo* imaging in infected cells and/or zebrafish embryos (Bernut et al., 2017).

In mycobacteria that produce and elaborate GPL on the bacterial surface, the synthesis of these lipids inside the cytosol and the transport across the plasma membrane is coupled and relies on a set of biosynthetic enzymes and lipid transporters, which form a large complex at the plasma membrane (Deshayes et al., 2010). It was previously demonstrated that the transmembrane protein MmpS4 is necessary for the proper complex formation of the GPL biosynthesis/transport machinery in *M. smegmatis* (Deshayes et al., 2010). On the other hand, the efflux pump protein MmpL4b was implicated in the transport of GPL in *M. abscessus* (Medjahed and Reyrat, 2009) and, more recently, it was shown that both MmpL4a and MmpL4b are required for the export of GPL in this mycobacterium (Bernut et al., 2016). Exploiting the genetic tools produced in this study, we inactivated the *mmpL4a*-*mmpL4b* gene couple and reproduced several of the phenotypes associated with the R colony morphotype of *M. abscessus*, including the loss of GPL. In addition, by replacing the endogenous promoter of *mmpS4* with an acetamide inducible promoter that is prone to poor repression in the absence of the inducer acetamide, we unintentionally obtained a low-level GPL producer that still exhibited phenotypes associated with the GPL-deficient rough variant of *M. abscessus*, notably aggregation and cord formation. Since genetic alterations within the GPL biosynthetic locus preceding R colony onset in clinical isolates were previously reported (Park et al., 2015), we further exploited this hybrid mutant that produces low GPL levels and exhibiting a R morphotype to investigate its virulence attributes using zebrafish embryos.

Since the R form of *M. abscessus* is considered more aggressive than the S form as it is often associated with more severe infections and decline of pulmonary functions in patients (Jönsson et al., 2007; Catherinot et al., 2009), we recently adapted the zebrafish infection model to understand why this is the case (Bernut et al., 2014, 2015, 2017). Using this animal model, we could explain the hypervirulent phenotype of the R through our observations that the R form has the ability to form serpentine cords, which serve as an efficient mechanism of immune evasion allowing this form to thrive extracellularly causing rapid larval death (Bernut et al., 2014, 2015, 2017; Halloum et al., 2016). However, the short duration of the zebrafish infection experiment, which lasts for no longer than 2 weeks and the low CFU with which the larvae are injected have been barriers to study the dynamics of the S-to-R transition as it occurs in real time *in vivo*. Using a set of *M. abscessus* strains exhibiting high (S strain), low (*mmpS4*::pUX3) or no (R strain) GPL, we further confirm the impact of the GPL profile on pathogenicity of *M. abscessus* and demonstrate that the presence of even low levels of this lipid strongly impacts on larval killing of a cord-forming R variant. It is, however, noteworthy that data obtained in zebrafish cannot necessarily be translated directly to human disease as embryos present disadvantages over mammalian models, including important anatomical differences as well as the lack of adaptive immunity in the early development stages, thereby affecting the outcome of the infection. Whereas embryos appear also more adapted to study acute infection with *M. abscessus*, the effect of the GPL profile on the chronic stages of the disease would be better modeled in other mammalian models such using immuno-compromised mice.

The same *M. abscessus* strains were also used to demonstrate a positive correlation between GPL production and hydrophilicity of *M. abscessus*. These results are analogous to those reported recently to demonstrate the implication of hydrophobicity in the evolution of *M. tuberculosis* pathogenicity, mainly related by the increased proportion of less polar lipids in the outer membrane (Minnikin et al., 2015; Jankute et al., 2017). Among all the strains tested, *M. kansasii* Hauduroy was the most hydrophilic strain due to the presence of large amounts of multiglycosylated phenolic glycolipids and lipooligosaccharides and restricted amounts or absence of important apolar lipids that characterize *M. tuberculosis* such diacyl trehalose, pentacyl trehalose or sulfoglycolipids. *M. abscessus* S, although being less hydrophilic than *M. kansasii*, was significantly more hydrophilic than *M. abscessus* R. In contrast, low GPL expression in *mmpS4*::pUX3 was associated with an intermediate phenotype. Changes in the cell wall associated lipids have been proposed to enhance the capability for aerosol transmission of *M. tuberculosis*. Whether this holds true for *M. abscessus, i.e*. that the hydrophobic R strain has acquired an increased propensity for aerosol transmission compared to the hydrophilic S strain is tempting. Supporting this view, epidemiological studies have documented the presence of the R variant in acute respiratory failure (Catherinot et al., 2009) and a significant association between an R phenotype and chronic colonization of the airways in CF patients (Jönsson et al., 2007). The possibility of aerosol transmission has recently been raised in world-wide surveys of *M. abscessus* infections (Bryant et al., 2013, 2016) but the possible link between this mode of transmission with the colony morphology/hydrophobicity has not been addressed and requires additional investigations. Since the evolutionary transformation from a hydrophilic, environmental, low-pathogenicity *M. abscessus* S to a hydrophobic, virulent *M. abscessus* R is particularly definite, the distinction between S and R variants should be better addressed and taken into account in epidemiological studies as this may help to better understand/anticipate transmission of *M. abscessus* among human populations that are exposed or vulnerable to *M. abscessus* infections.

## Author contributions

AV conceived the idea of the project, conducted experiments, analyzed the data and wrote the paper. AG conducted experiments, analyzed the data. CD conducted experiments, analyzed the data. EG analyzed the data and participating in writing the paper. LK conceived the idea of the project, analyzed the data and wrote the paper.

### Conflict of interest statement

The authors declare that the research was conducted in the absence of any commercial or financial relationships that could be construed as a potential conflict of interest.
